# CRISPRedict: a CRISPR-Cas9 web tool for interpretable efficiency predictions

**DOI:** 10.1093/nar/gkac466

**Published:** 2022-06-07

**Authors:** Vasileios Konstantakos, Anastasios Nentidis, Anastasia Krithara, Georgios Paliouras

**Affiliations:** Institute of Informatics and Telecommunications, NCSR Demokritos, Patr. Gregoriou E & 27 Neapoleos Str, 15341 Athens, Greece; Institute of Informatics and Telecommunications, NCSR Demokritos, Patr. Gregoriou E & 27 Neapoleos Str, 15341 Athens, Greece; School of Informatics, Aristotle University of Thessaloniki, 54124 Thessaloniki, Greece; Institute of Informatics and Telecommunications, NCSR Demokritos, Patr. Gregoriou E & 27 Neapoleos Str, 15341 Athens, Greece; Institute of Informatics and Telecommunications, NCSR Demokritos, Patr. Gregoriou E & 27 Neapoleos Str, 15341 Athens, Greece

## Abstract

The development of the CRISPR-Cas9 technology has provided a simple yet powerful system for genome editing. Current gRNA design tools serve as an important platform for the efficient application of the CRISPR systems. However, most of the existing tools are black-box models that suffer from limitations, such as variable performance and unclear mechanism of decision making. Here, we introduce CRISPRedict, an interpretable gRNA efficiency prediction model for CRISPR-Cas9 gene editing. Its strength lies in the fact that it can accurately predict efficient guide RNAs—with equivalent performance to state-of-the-art tools—while being a simple linear model. Implemented as a user-friendly web server, CRISPRedict offers (i) quick and accurate predictions across various experimental conditions (e.g. U6/T7 transcription); (ii) regression and classification models for scoring gRNAs and (iii) multiple visualizations to explain the obtained results. Given its performance, interpretability, and versatility, we expect that it will assist researchers in the gRNA design process and facilitate genome editing research. CRISPRedict is available for use at http://www.crispredict.org/.

## Graphical Abstract

**Graphical Abstract:**
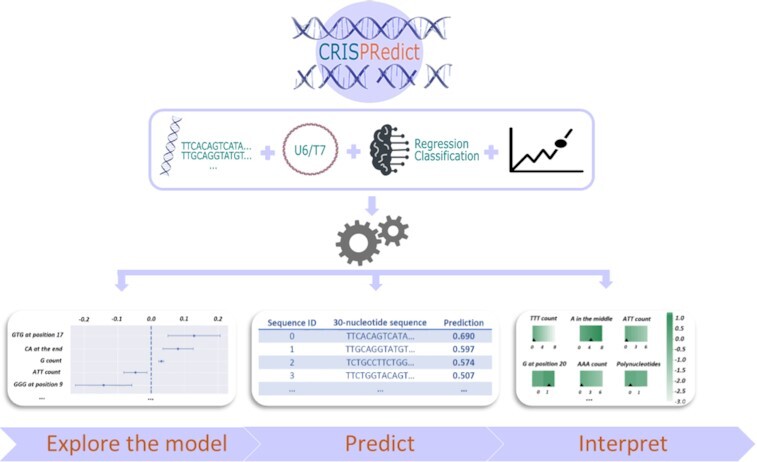
CRISPRedict is an interpretable gRNA efficiency prediction model for CRISPR-Cas9 gene editing. It can accurately predict efficient gRNAs—with equivalent performance to state-of-the-art tools—while being a simple linear model.

## INTRODUCTION

The CRISPR-Cas9 system has revolutionized the field of genome editing and promises the ability to study genetic interactions at their origin and the opportunity to cure severe inherited diseases. Compared with previous gene-editing tools, such as zinc-finger nucleases (ZFNs) and transcription activator-like effector nucleases (TALENs), which bind to a specific DNA sequence by protein-DNA recognition, the CRISPR-Cas9 system identifies target sites by the complementarity between the guide RNA (gRNA) and the DNA sequence, which is less expensive and time-consuming, as well as more precise and scalable.

To effectively use the CRISPR-Cas9 system for gene editing, researchers need to identify target sites that can be cleaved efficiently and for which the candidate gRNAs have little or no cleavage at other genomic locations. Specifically, an ideal gRNA should maximize on-target activity (guide efficiency) while minimizing potential off-target effects (guide specificity). Balancing these two requirements can be a challenging task and, as a result, significant effort in recent years has been focused on developing computational tools to assist in the design of gRNAs (1–4).

However, currently available tools still have some limitations, such as their variable performance (5). In particular, they cannot robustly predict experimental success on new gene editing tasks that may deviate from the data the model was trained on. In addition, most of the popular tools are black-box models that do not provide intrinsically explainable predictions. Therefore, the specific factors that determine on-target activity remain largely unexplored. Finally, many of the current gRNA design tools (e.g., *TUSCAN*(6)) are not implemented as a web server but only provide source code to obtain their predictions. A brief overview of the existing activity prediction web servers is provided in Table 1.

**Table 1.** List of gRNA activity prediction web servers

Name	URL	Input	Guide Promoter	Interprets Predictions	Provides Classification
DeepCRISPR (9)	http://www.deepcrispr.net/	N/A	N/A	N/A	N/A
DeepSpCas9 (3)	http://deepcrispr.info/DeepSpCas9/	3000 gRNAs per input	U6	No	No
DeepHF (4)	http://www.deephf.com/#/cas9	Single target sequence (1k)	U6, T7	No	No
Azimuth 2.0* (2)	https://crispr.ml/	300/500 gRNAs per input	U6	No	No
CRISPRscan (8)	https://www.crisprscan.org/	Single target sequence (10k)	T7	No	No
CRISPOR (23)	http://crispor.tefor.net/	Single target sequence (25k)	U6, T7	No	Yes
TSAM (12)	http://www.aai-bioinfo.com/CRISPR/	N/A	N/A	N/A	N/A
sgDesigner (13)	http://crisprdb.org/wu-crispr/	Single target sequence (30k)	U6	No	Yes
**CRISPRedict**	http://www.crispredict.org/	Unlimited sequences per file	U6, T7	Yes	Yes

The maximum accepted length of a single target sequence is provided in parentheses. The tool described in this work is marked in bold.

*Azimuth 2.0 is also available at https://portals.broadinstitute.org/gppx/crispick/public. N/A: Not available.

Here, we introduce *CRISPRedict*, a simple and interpretable linear model that accurately predicts guide activities. Comprehensive evaluation using independent data demonstrated that *CRISPRedict* achieves equivalent performance to the currently most accurate tools and outperforms many others (7). Moreover, it exhibits a robust performance for different types of data, illustrating its general applicability under different experimental conditions.

Implementing the trained model as a user-friendly web server, *CRISPRedict* not only delivers quick and accurate predictions, but also provides visualizations to explain the obtained results. These results can then be used to guide genome editing experiments and make plausible hypotheses for further investigation. Finally, in addition to the popular choice of regression models, the web server includes a classification variant of *CRISPRedict* that provides the additional benefit of a clear threshold for labeling gRNAs. In the following sections, we give a quick overview of the server and provide case studies to demonstrate its use.

## MATERIALS AND METHODS

This section summarises the approach that we followed to generate the prediction models and implement them as a web server. Detailed information regarding the training and evaluation process is provided in a separate study (7).

### Model construction

A U6 and a T7 variant of *CRISPRedict* was created to improve the prediction ability of the model under different gRNA expression conditions. Each variant was trained on a different dataset, using a similar modeling pipeline. The pipeline is presented briefly below.

First, we defined the initial feature set using sequence characteristics that have been shown to influence cleavage efficiency (2,5,8). This includes overall and position-specific nucleotide composition, as well as features that reflect the structural properties of the guide sequence. Then, we applied a multi-step feature selection strategy to arrive at a minimal and relevant-only feature subset. We also evaluated a set of different algorithms at each step and selected the appropriate one for each task. For each case, we chose the model that balances best between performance and complexity.

Having determined the set of features and the appropriate algorithms, we trained one regression and one classification model for each task. In particular, we trained a binomial regression and a linear regression model to predict the percentage of efficient edits for the U6 and T7 variant, respectively. Moreover, we used the same (i.e. 28 and 25) features to train two logistic regression models by labeling the top 20% and bottom 20% gRNAs as efficient and inefficient, respectively. Therefore, we created four models for the four different tasks.

### Performance evaluation

We evaluated *CRISPRedict* using twelve published datasets, derived from studies from independent laboratories (7). These datasets included gRNAs expressed with the U6 or the T7 promoter and represented guide efficiency using either a continuous or a discrete variable. Using these datasets, we compared *CRISPRedict* against eight state-of-the-art gRNA design tools, including *DeepCRISPR* (9), *DeepCas9* (10), *DeepSpCas9* (3), *DeepHF* (4), *CRISPRLearner* (11), *Azimuth 2.0* (2), *TSAM* (12) and *sgDesigner* (13).

Our thorough evaluation demonstrated that *CRISPRedict* achieves an equivalent performance with the currently most accurate tools. Moreover, it exhibited the most robust performance for both U6 and T7 datasets, illustrating its applicability to tasks under different conditions.

**Figure 1.**
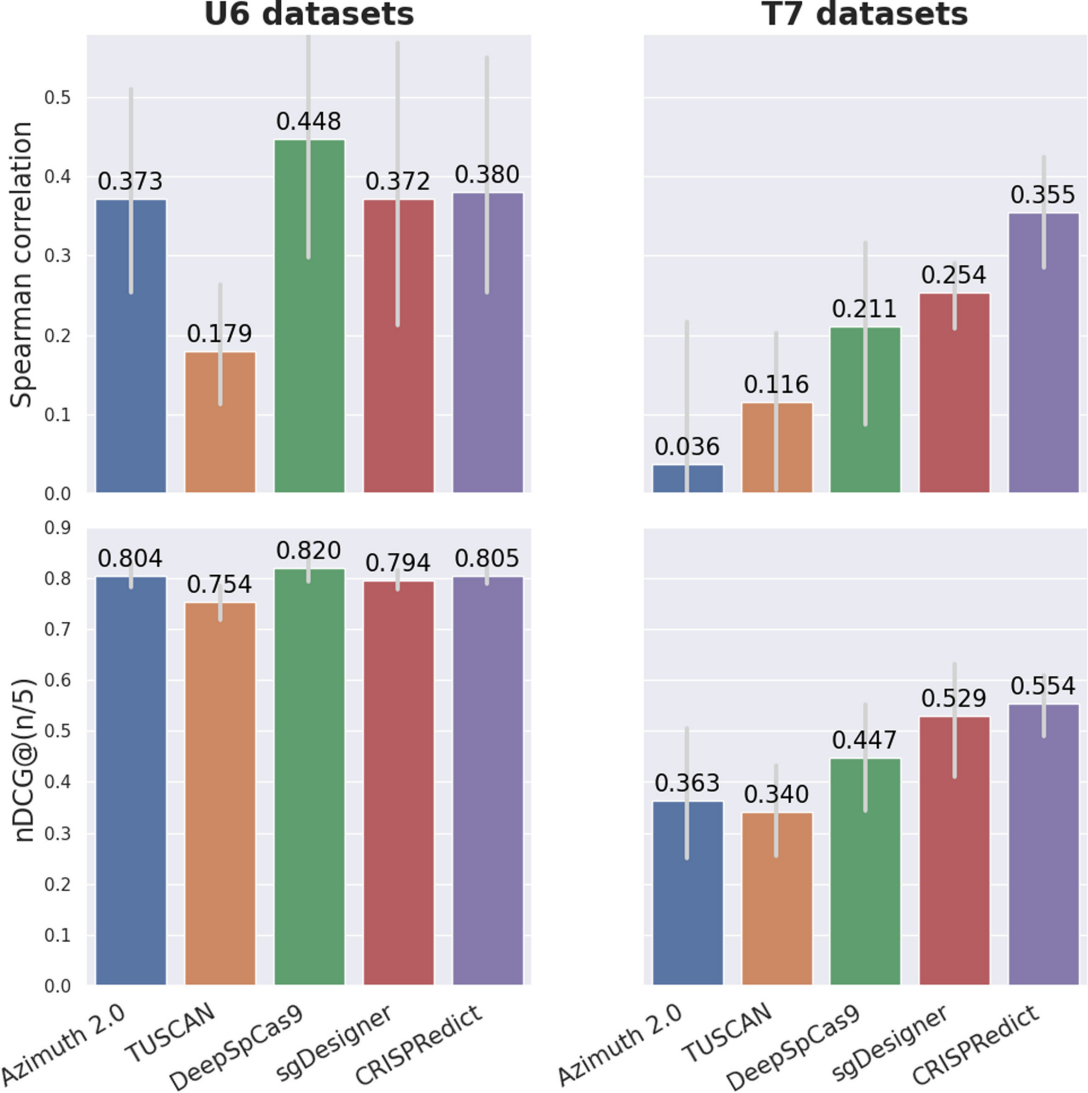
Comparison of the general performance of the CRISPRedict model. Each bar demonstrates the mean performance of a model across all U6 or T7 datasets, using a single evaluation metric. nDCG: normalized discounted cumulative gain, *n*: number of total samples.

A summary of the results among selected models can be seen in Figure 1. In particular, we chose the most popular (*Azimuth 2.0*) and the most accurate deep learning (*DeepSpCas9*) and machine learning methods (*sgDesigner*) that were also included in previous comparisons (3,5,13–14). We also evaluated *TUSCAN* (6), a Random Forest (RF)-based tool that is trained on sequencing data using 63 features and provides scalable predictions. We selected two metrics for the presented evaluation; namely Spearman correlation and normalized discounted cumulative gain (nDCG) that capture the general performance and the top-ranking ability of the models, respectively. We used these metrics to compare the five selected models on four U6 and four T7 datasets. The detailed results can be found in the Supplementary material (Supplementary Table S1).

As shown in Figure 1, *CRISPRedict* outperforms the chosen models on the T7 datasets and has the second-best performance on the U6 datasets. In addition, it achieves a more robust performance (smaller variance) across all datasets.

### Server implementation

*CRISPRedict* is written in Python 3 and uses the Streamlit library for the web application. Files are parsed and written using Biopython (15) and Pandas (16). The visualizations are generated using the Matplotlib (17) and Seaborn (18) plotting libraries. All models are implemented using the Statsmodels (19) library. The SHAP library (20) is also used to obtain explainable predictions, along with custom Python scripts.

## RESULTS

In this section, we present an overview of the server’s capabilities. We also describe case studies that utilize the different functionalities in order to further demonstrate the general use and applicability of the web server.

### Web server usage

**Figure 2.**
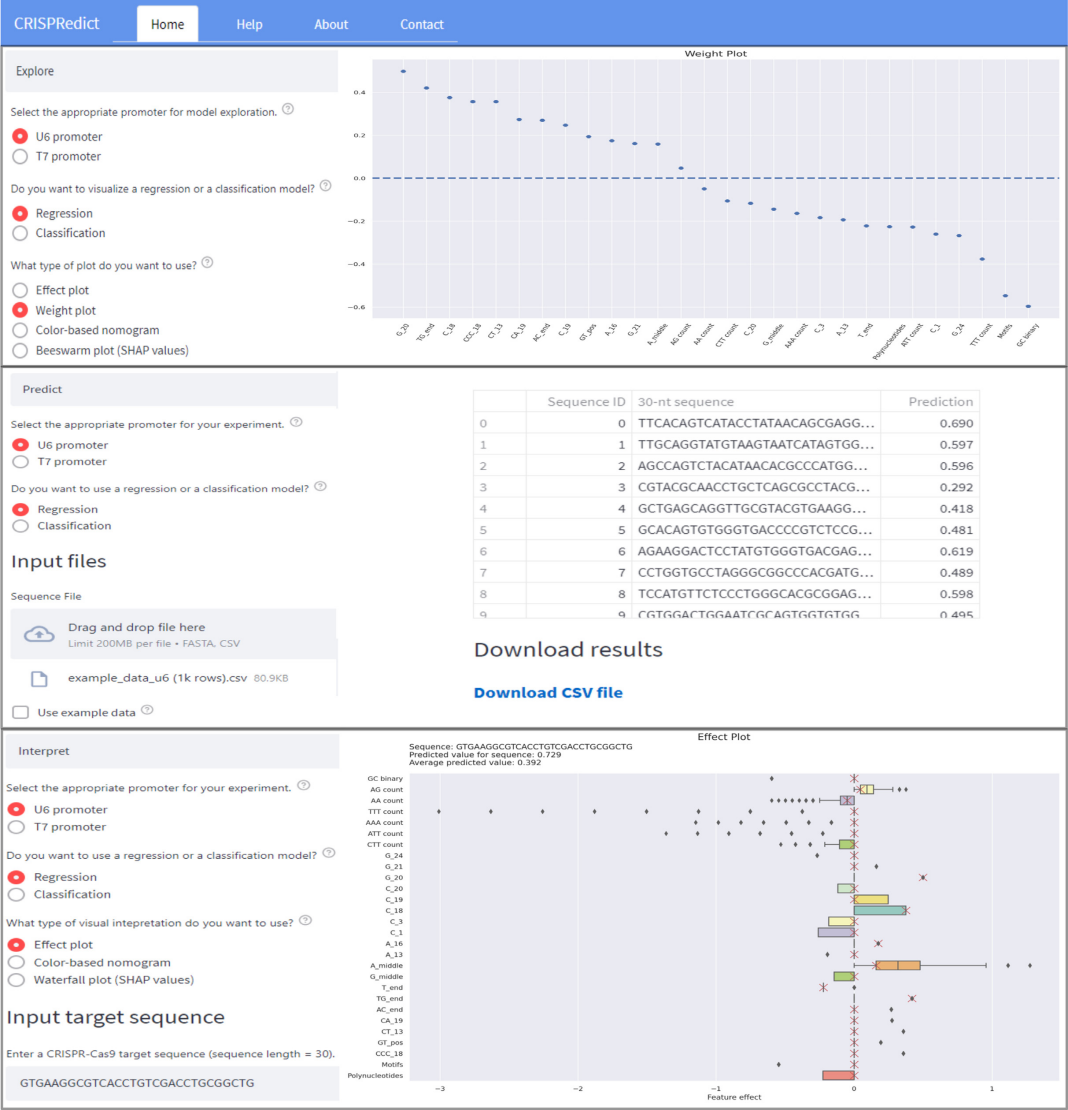
Overview of the CRISPRedict webserver. Illustrates the different functionalities with example input/output.

*CRISPRedict* offers three different modes of operation (Figure 2). The ‘Explore’ mode provides visualizations to explore the features and parameters of the predictive models. Its purpose is to familiarize the users with the provided models before choosing the appropriate one for their experiments. The second mode (‘Predict’) can be used to obtain guide efficiency predictions for one or more target sequences. It accepts 30-nucleotide (nt) sequences given in a FASTA or comma separated values (CSV) file. Instructions regarding the expected file format are provided along with example data for demonstration. The results are displayed in a sortable table that can be downloaded for future use. Finally, the ‘Interpret’ mode provides visualizations that help interpret the predictions obtained for a particular sequence. It accepts a 30-nt sequence as input with an example sequence already provided. Information regarding sequence formatting is also presented, along with a way to generate random valid sequences. The resulting visualizations are provided as downloadable images. Features are also displayed in a table below the obtained results. All the described modes include configuration options and tool-tips for guidance. A help page is also provided with an example input/output for each category.

### Case study 1: exploring trained models

**Figure 3.**
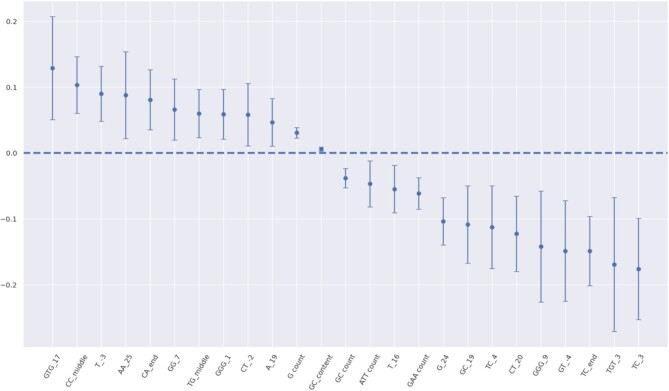
Weight plot of the trained T7 regression model. Weights are displayed as points and the 95% confidence intervals as lines. The x-axis includes position-dependent features, such as the presence of adenine in position 19 (A_19), as well as global features, such as the total number of guanines (G count).

Before choosing the appropriate model for a given task, it is crucial to understand its parameters and how they might influence the final result. For this reason, the ‘Explore’ mode provides various visualizations to investigate the features of each predictive model. An example output is shown in Figure 3.

In the case of the T7 regression model, we observe that many of the model’s weights exhibit great variability. However, features like ‘G count’ and ‘GC count’ have a more reliable, albeit lower, contribution to the final predictions. In contrast, the U6 variant of *CRISPRedict* consists of more stable weight parameters (Supplementary Figure S1), attributed to the larger dataset on which it has been trained.

### Case study 2: predicting multiple sequences

In this case, we examine how researchers can use *CRISPRedict* predictions and the knowledge about on-target activity in their experiments. To accomplish this, we present a study that involves the treatment of a genetic disorder. In particular, we use *CRISPRedict* to select the appropriate gRNAs and illustrate how the information provided by the server can inform the experimental design. Similar to Peng *et al.* (12), we use retinitis pigmentosa (RP) as an example.

Yu *et al.* (21) attempted to knockdown the *Nrl* gene to prevent retinal degeneration in a mouse model and suggested an adeno-associated virus (AAV)-based CRISPR-Cas9 system for gene disruption as a promising treatment option for patients with RP. To accomplish that, they designed five candidate gRNAs (denoted NT1 to NT5) against the mouse *Nrl* coding region. Among those, they selected NT2 for the *in vivo* study based on its higher ability to generate indels and lower predicted off-target potential.

**Figure 4.**
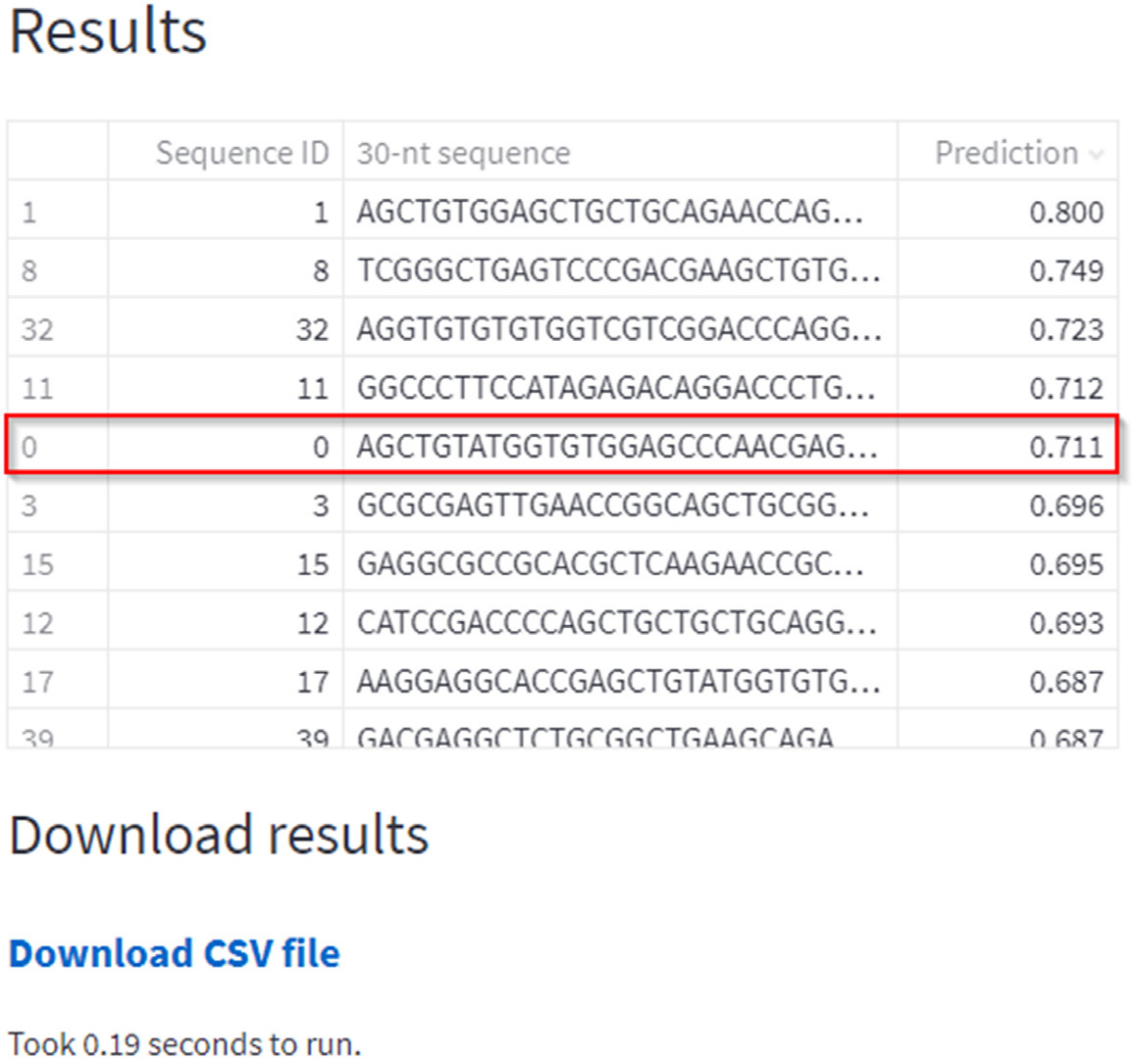
Prediction output for the 147 candidate gRNAs of the Yu *et al.* study. The output of the table has been sorted based on the predicted score in order to select the most efficient guide sequences. The NT2 gRNA that was selected by Yu *et al.* for the *in vivo* study is marked.

In our experiment, we used *CHOPCHOP* (1) to retrieve all the candidate sequences for *Nrl* gene knockdown in the mouse genome (mm39). In total, 147 potential spacer sequences were identified with the PAM 5′-NGG-3′. The cleavage efficiencies of the 147 candidate gRNAs were predicted by *CRISPRedict* and are shown in Figure 4.

We observe that *CRISPRedict* ranks the gRNA selected by Yu *et al.* (NT2) in the 5th position. Therefore, researchers could have identified the efficient gRNA by experimenting with the top five of the 147 possible sequences. Such a recommendation approach can save time and cost, without sacrificing accuracy and efficiency.

### Case study 3: interpreting efficiency predictions

We now highlight the ability of *CRISPRedict* to provide interpretable and explainable predictions for the CRISPR-Cas9 system. To accomplish that, we present a different case study involving again the treatment of a genetic disorder, namely X-linked chronic granulomatous disease (X-CGD). Specifically, we use the classification model of *CRISPRedict* to differentiate between efficient and inefficient gRNAs and explain their differences.

De Ravin *et al.* (22) used CRISPR-Cas9 to repair a mutation in the CYBB gene of CD34+ hematopoietic stem and progenitor cells (HSPCs) from patients with the immunodeficiency disorder X-CGD. Unlike our case study 2, the cutting site should be close to the mutation site in order to promote homology-directed repair (HDR) and correct the point mutation. Four guide sequences (gRNA1, gRNA2, gRNA3 and gRNA8) around the CYBB mutation site were evaluated, of which gRNA2 displayed maximal cutting efficiency.

**Figure 5.**
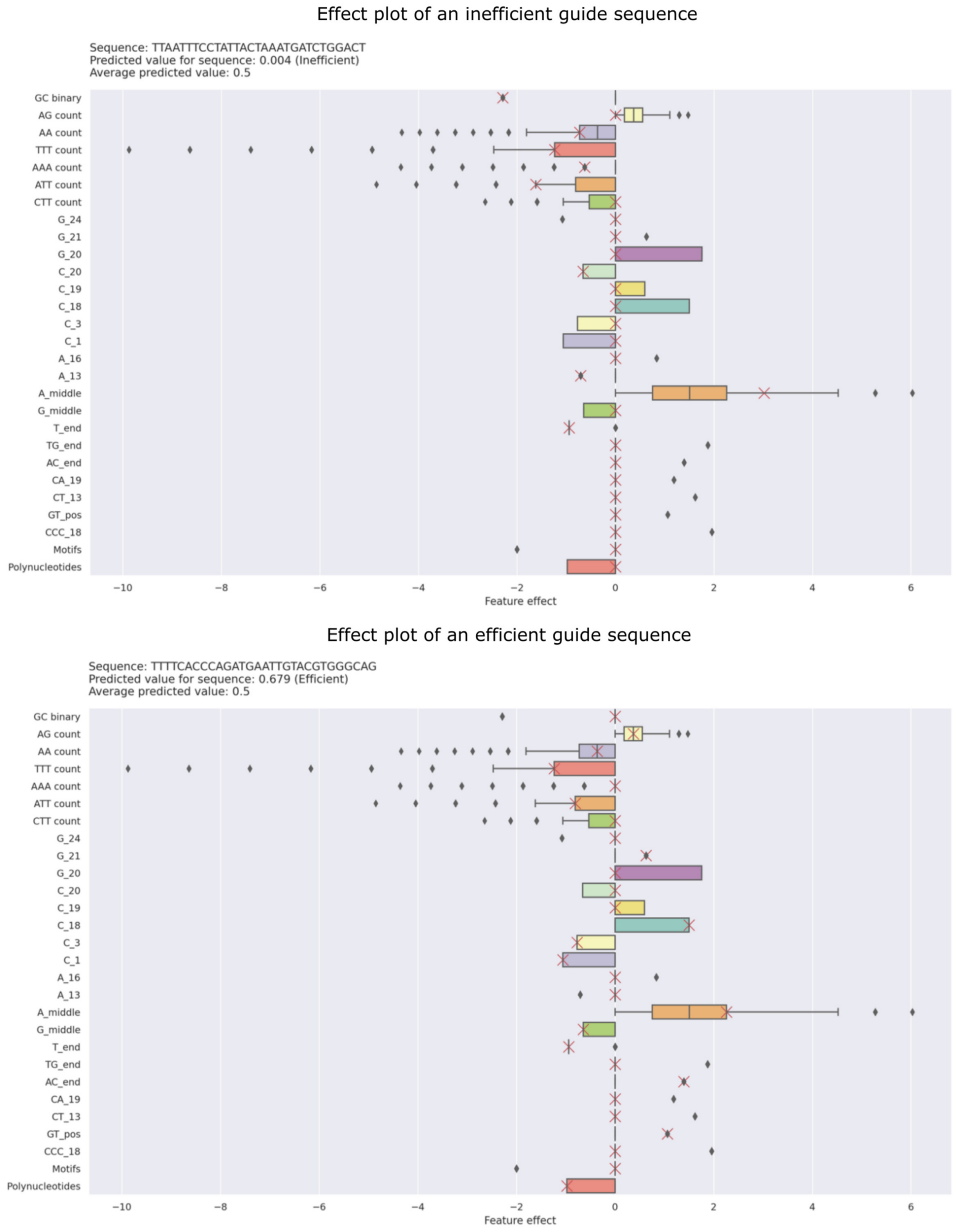
CRISPRedict output for an efficient and inefficient guide RNA. The effect plot shows the distribution of effects (= feature value times feature weight) across the training data using boxplots. The boxes contain the effect range for half of the data (25–75% effect quantiles). The horizontal lines extend to the ±1.5 inter quartile range (IQR), while the vertical lines are the median effect. The diamonds represent outliers. The crosses show the actual feature effects for the provided instance.

Similar to case study 2, we used *CRISPRedict* to predict the cleavage efficiency of each gRNA. However, we focused here on the most and least efficient guide sequence and used the ‘Interpret’ mode to illustrate their differences (Figure 5). In particular, the effect plot was used to demonstrate how the combination of model weights and feature values results in the final prediction. We chose this plot because it combines global (visualizing feature distribution) and instance-based (marking actual feature effect) information.

We observe that the predicted scores are 0.679 and 0.004 for the efficient and inefficient guide sequence, respectively. Thus, *CRISPRedict* differentiates the efficient and inefficient gRNAs by a large margin. In addition, the effect plots provide a direct interpretation for these predictions. For instance, the inefficient gRNA includes mostly features that negatively impact on-target activity, such as extreme GC content and increased TTT count, confirming the results of previous studies (5). Similarly, the efficient guide sequence mostly includes features with positive impact, such as the AG count and the number of adenines (As) in the middle of the sequence. Therefore, *CRISPRedict* not only differentiates between active and non-active gRNAs but provides visual explanations for its predictions.

## CONCLUSION

Due its simplicity and versatility, the CRISPR-Cas9 system has been widely adopted by the scientific community to target and modify the genomes of a vast array of cells and organisms. Computational gRNA design tools facilitate the efficient application and development of CRISPR systems. However, the existing models have several limitations, such as their variable accuracy and unclear mechanism of recommendation.

In this work, we have introduced *CRISPRedict*, an easy-to-use web tool for gRNA activity prediction. Having been extensively evaluated in terms of accuracy and robustness, *CRISPRedict* provides interpretable efficiency predictions with comparable performance to state-of-the-art design tools. Given its performance and versatility, we expect that it will greatly facilitate genome editing research. To ensure that *CRISPRedict* remains competitive and up-to-date, it will be periodically updated as new data become available.

## DATA AVAILABILITY

*CRISPRedict* is implemented in Python and is freely available as both web server and standalone software under the Apache 2.0 license. The web server can be accessed at http://www.crispredict.org/ without a login process. The standalone version can be downloaded from the GitHub repository: https://github.com/VKonstantakos/CRISPRedict.

## Supplementary Material

gkac466_Supplemental_Files
