# Supplementary material for: CRISPRedict: a CRISPR-Cas9 web tool for interpretable efficiency predictions
Source: Nucleic Acids Res. 2022 Jun 7;50(W1):W191–8. doi: 10.1093/nar/gkac466 (PMC9252759; doi:10.1093/nar/gkac466)
Supplement: gkac466_Supplemental_Files [file gkac466_supplemental_files.zip › Supplementary Figure 1. Weight plot of the trained U6 model.pdf]

Supplementary Figure 1

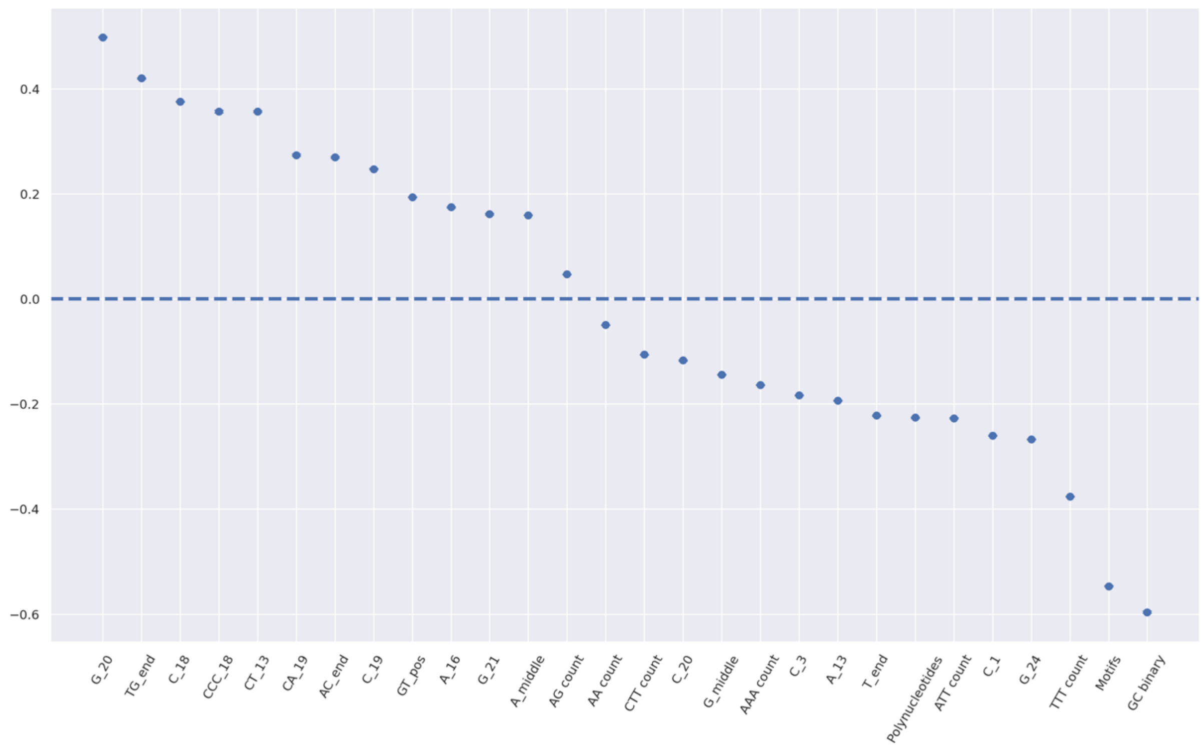

Supplementary Figure 1: Weight plot of the trained U6 regression model. The x-axis includes position-dependent features, such as the presence of guanine in position 20 (G_20), as well as global features, such as the total number of adenine-guanine dinucleotides (AG count).
